# Crowding-Out or Crowding-In: Government Health Investment and Household Consumption

**DOI:** 10.3389/fpubh.2021.706937

**Published:** 2021-06-11

**Authors:** Hao Cheng, Yu-Peng Zhi, Zi-Wei Deng, Qing Gao, Rui Jiang

**Affiliations:** ^1^College of Economics and Management, Nanning Normal University, Nanning, China; ^2^Business School, Guangxi University, Nanning, China; ^3^School of Economics and Management, Guangxi Normal University, Guilin, China; ^4^Graduate School, Nanning Normal University, Nanning, China; ^5^School of Labor and Employment Relations, University of Illinois at Urbana-Champaign, Champaign, IL, United States

**Keywords:** crowding-out, crowding-in, government health investment, household consumption, Sobel-Goodman mediation tests

## Abstract

This paper explores the relationship of government health investment and household consumption by applying a panel fixed effects model and Sobel-Goodman mediation tests to inland Chinese provinces. The empirical results highlight that government health investment has a crowding-in effect and can thus promote household consumption. Furthermore, the promotion effect on non-medical health consumption is greater than that on medical health consumption. The promotion effect of government health investment on rural household consumption is higher than that on urban household consumption, and the promotion effect on household consumption for northern provinces is higher than that in southern provinces. This heterogeneous effect is closely related to the difference between urban and rural development; and the economic levels of the northern and South regions. The mediation tests found that government health investment mainly promotes regional economic growth, and then increases household consumption. In the economic and social development process, the government should implement more effective medical and health care measures to increase social medical and health investment to improve the consumption level of households.

## Introduction

The main purpose of this paper is to explore whether there is a crowding-out effect or crowding-in effect between government health investment and household consumption. If there is an effect, is it crowding-in or crowding-out? In recent years, with the development of the world economy, China's economy has also improved, which is reflected in the improvement of the country's household consumption. According to the health data of the China Statistical Yearbook, the government health investment and disposable income of Chinese residents are increasing each year, and the correlation between government health investment and household consumption is more intuitive in the economic development process. With the economic growth, China's medical and health system has been constantly reformed to meet the needs of residents and the huge demand for health security resulting from economic growth (1). Therefore, the government's health investment has triggered the transformation of the medical and health industry and reduced the household medical and health consumption to a certain extent, which is reflected in the changes in household consumption in the past 10 years. Studies have shown that increases government health investment may increase household consumption (or increase non-medical and health consumption consumption), mainly because an increase in government health investment can improve the specialization of medical equipment and services in society overall; that is, it reduces household medical health consumption by improving social welfare, and then expanding consumption in other aspects (2). Furthermore, under the more specialized medical service system, the health of residents can be improved, and a lager labor force and more efficient working time will be created, which can realize rapid economic growth and improve the per capita disposable income of residents, and then affect the consumption of residents (3). Therefore, there is a correlation between government health investment and household consumption, which has a significant impact on household consumption. This study is helpful for policy-makers to pay attention to the potential relationship between government health investment and household consumption (including non-medical health consumption and medical health consumption). In the economic development process, the government should adopt more active medical policies to reduce the medical and health consumption of residents, and increase non-medical health consumption.

For developing countries and low-income countries, the financial burden caused by health consumption is an urgent problem. Public investment in health in these countries is relatively low, there are no proper safety net mechanisms, and public health systems are poor quality. The proportion of residents' medical health consumption is high, and the consumption expenditures are large. The effect of the increase in medical health consumption is reflected in the decrease in the share of non-food items such as education, entertainment and clothing. Poor households in regions with high public health expenditures have higher proportions of expenditures and mainly reduce their expenditures on education while poor households in regions with low public health expenditures have reduced most of their non-food consumption (4). With global population aging becoming increasingly more serious, there is an “inverted U-shaped” relationship between government investment in healthy aging and economic growth. Government investment in healthy aging will increase the healthy human capital held by the elderly and promote economic growth, but it will also squeeze out the accumulation of material capital and hinder economic growth. Economic growth has a direct impact on household consumption, so the impact of government health investment on household consumption is a “double-edged sword.” While, it can promote an increase in consumption, it may inhibit household consumption. This also explains the mechanism of government health investment on household consumption: government health investment improves the labor supply in the market by increasing the proportion of healthy human capital and increase the economic growth rate to expand household consumption. In addition, government health investments can also reduce per capita consumption by crowding out physical capital accumulation when the economy is in a steady state.

**Figure 1:**
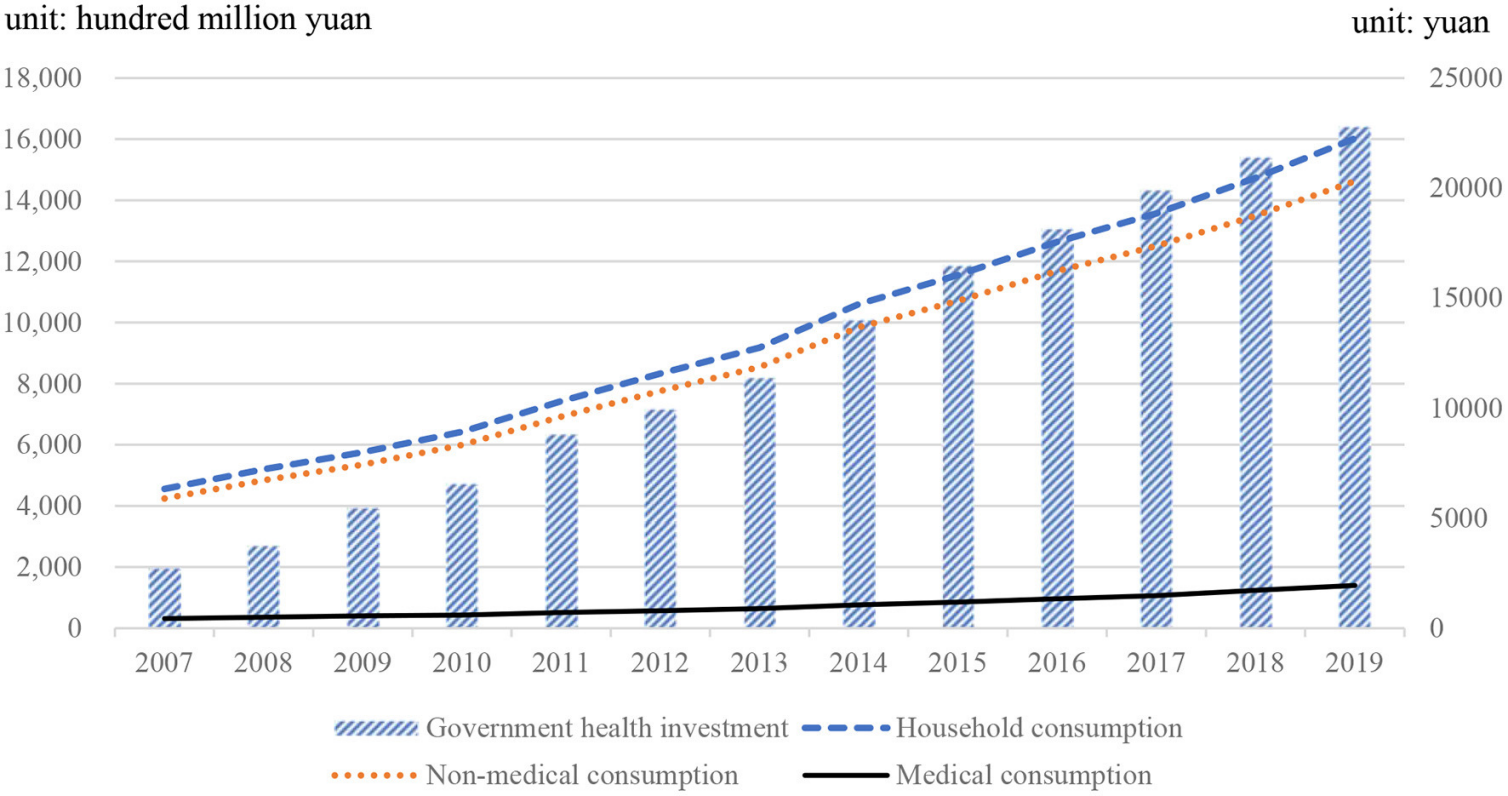
Changes in government public health investment and household consumption. Data source: China Statistical Yearbook.

In the economic development process, the correlation between government health investment and household consumption can not be ignored. According to the classification of China Statistical Yearbook, consumer spending is composed of non-medical consumption and medical consumption. Research shows that government health investment has a positive effect on economic development, but there are also problems such as relatively high population density and insufficient household consumption. Although the living standards of urban and rural residents in China have basically reached a well-off society, the consumption of basic needs is still relatively high. Affected by housing and other factors, the initiative of urban and rural residents in health investment is obviously insufficient. In addition, regarding future consumption, and household consumption, there is a co-integration relationship between government health investment and consumption. The imbalance between them will be corrected in the short term. Figure 1 shows the relationship between household consumption and government health investment. As can be seen from Figure 1, China's government health investment has increased year by year. During the study period, it has increased by 1442.187 billion yuan, reaching 1641.762 billion yuan in 2019, which is 8.23 times the government health investment in 2007 (195.575 billion yuan). This provides protection for national health. With the continuous improvement of the consumption level of residents, the amount of medical consumption expenditure is also increasing year by year, which is reflected in the growing gap between household consumption and non-medical consumption.

There was no parallel in history to the international background of the outbreak of coronavirus disease 2019 (COVID-19), where economic policy uncertainty in various countries has been higher than ever (5). The scale of the “home economy” has expanded rapidly, and consumption in restaurants, tourism, and sports fields has been suppressed, which has had an unprecedented impact on the effect of China's health investment on household consumption. Although existing studies have included different periods and different regions, the conclusion shows that government health investment has both positive and negative effects on household consumption, and the specific effect is uncertain. This paper considers a panel fixed effect model to explore the crowding-in or crowding-out relationship between government health investment and household consumption in inland provinces of China. In addition, we also use the Sobel-Goodman mediation test to further verify the mechanism between the two.

The remainder of the paper is structured as follows. Section Literature reviews the existing literature, and section Government health investment and household consumption model presents the model designed in this paper. Section Methodology describes the econometric approach employed in this paper. Section Data describes the data. Section Empirical results presents the findings of the study. Section Conclusions offers concluding remarks.

## Literature Review

Modern macroeconomic models suggest conflicting effects of government spending on private economic activities (6). In a general model of consumption, there may be a crowding out effect or crowding in effect between government spending and private consumption (7), and we should allow for the direct effect of government purchases of goods and services on a consumer's utility (8, 9). Therefore, the effect of government purchases of goods and services on household consumption is controversial theoretically and empirically (10). In the traditional IS-LM model, the increase in government spending leads to a heavier tax burden on residents, thus reducing their disposable income and consumption (11, 12), which shows that public consumption is a close substitute for private consumption (13). Barro (14) stressed that public spending has a substitution effect on private consumption. Basu and Kimball (15) argued that in a New Keynesian model with sticky prices, the negative wealth effect is so strong that output also decreases when public spending on consumption increases. Ahmed (16) used an intertemporal substitution model to estimate the effects of government consumption using UK data. He finds that government consumption tends to crowd out private consumption. Ho (17) extended the existing literature to panel data for OECD countries and used panel cointegration methods to find that there is a significant degree of substitutability between government spending and private consumption when real disposable income is included. Amano and Wirjanto (9) applied a relative price approach to estimate the intratemporal elasticity of substitution between government spending and private consumption. They found that in the US, the elasticity of substitution between government spending and private consumption is ~0.9. Based on the analysis of group samples from 145 countries, Furceri and Sousa (10) found that government expenditures have a significant crowding out effect on private consumption, and there are regional differences in this negative effect. Kormendi (18) explores the permanent-income approach and finds a significant degree of substitutability between private consumption and government spending for the United States.

However, many studies have shown that government spending will crowd in household consumption (19). By modifying the Keynesian model to allow the rule of thumb consumers to coexist with traditional unlimited vision Ricardo consumers, Jordi et al. (20) find that there is a positive effect of government spending on consumption. Deficit reductions engineered through cuts in public investment severely affect private capital accumulation and growth prospects (21). Karras (22) examined the impact of government expenditure on private consumption. The results show that increased government expenditures tend to promote private consumption, and the two are complementary rather than substitutes. In other words, government spending tends to raise the marginal utility of private consumption. Nieh and Ho (23) used data from OECD countries and found that there is a co-integration relationship between private consumption and government expenditures, and both of them are complementary, which indicates that there is no crowding-out effect of expansionary government expenditures on private consumption. Funashim and Ohtsuka (6) find that considering the spatial spillover effect and regional differences, government expenditures have a certain foundational effect on private consumption. Khan et al. (24) revealed that government spending have positive impact on private consumption, and government spending has a positive impact on private consumption, and government spending is a very good instrument to boost the economy and encourage aggregate demand in China during a recession. Tenhofen et al. (25) found that government spending and private consumption are positively related to some extent, and the expected expenditure impact has a significant impact on output when the impact is realized.

In the actual health consumption process, in addition to the consumption of residents themselves, government health investment also needs to pay for their remaining health consumption. Based on the perspective of bounded rational persons, insurance can affect household consumption under certain conditions. International research has considered the impact of government health consumption on household consumption from the perspective of public health insurance. Edson et al. (26) took Mexican family medical services as the breakthrough point and found that the prevalence of outpatient consultation and drug use was the highest in the medical service consumption category (11.2%), while the proportion of health service consumption of uninsured families (8.4%) was relatively low. Under the financial protection policy of Mexico, the variety of medical services has been increasing, and the poor have achieved gradually increased access to health care. Through exploring the experience of public health insurance in mitigating adverse effects related to health shocks, Liu (27) finds that the Chinese family labor supply is an important safeguard mechanism against health shocks but that obtaining public health insurance helps families maintain high investments in children's human capital while facing negative health shocks, that is, to ensure family education consumption. However, the view that government health investment can promote household consumption is not always confirmed. By comparing the household survey data before and after the initiation of the community health insurance programme in Ethiopia, Debebe et al. (28) found that the purchase of health insurance can reduce the vulnerability of families to self-financed medical health consumption, but there is no actual evidence that participation in the health insurance programme will have a positive impact on household consumption.

In summary, the research results based on different countries and different perspectives show that there is a correlation between government health investment and household consumption. However, it is unknown whether government health investment has a crowding-in effect or crowding-out effect on household consumption. Therefore, this paper will focus on the relationship between the two in-depth.

### Government Health Investment and Household Consumption Model

In this paper, we assume a representative consumer who can live indefinitely. The utility function is in the form of CRRA, and the specific form is:

(1) $$\begin{array}{l} \text{U}=\int_{0}^{\infty }\frac{1}{{\left(1+\rho \right)}^{t}}\frac{{\left(C_{t}\right)}^{1-\theta }}{1-\theta }dt \end{array}$$

Here, θ is the relative risk aversion of consumers (θ > 0, θ ≠ 1), ρ is the discount factor (ρ > 0); and $\frac{{\left(C_{t}\right)}^{1-\theta }}{1-\theta }$ is the CRRA function, representing the consumer's current utility in period *t*. Since there are two types of consumption studied in this paper and their utility is also different, we rewrite household consumption (*C*_*t*_) into the following form:

(2) $$\begin{array}{l} C_{t}={\left(c_{t}\right)}^{\alpha }{\left({\text{M}}_{t}\right)}^{\beta }{\left(G_{t}\right)}^{\gamma } \end{array}$$

Here, *c*_*t*_ is non-medical health consumption, M_*t*_ is medical health consumption, and *G*_*t*_ is government health investment. α, β and γ represent the correlation coefficients between non-medical health consumption, medical health consumption and government health investment, respectively.

This consumer pursues utility maximization, and the maximized utility function is:

(3) $$\begin{array}{l} \underset{c_{t},m_{t}}{\min}\;U=\int_{0}^{\infty }\frac{1}{{\left(1+\rho \right)}^{t}}\frac{{\left[{\left(c_{t}\right)}^{\alpha }{\left({\text{M}}_{t}\right)}^{\beta }{\left(G_{t}\right)}^{\gamma }\right]}^{1-\theta }}{1-\theta }dt \end{array}$$

(4) $$\begin{array}{l} \text{subject to }c_{t}+{\text{M}}_{t}\leq W_{t}-T_{t} \end{array}$$

Here, in the constraint condition, *W*_*t*_ is the income of consumers in period t, *T*_*t*_ is the tax paid by consumers in phase t, and *W*_*t*_−*T*_*t*_ is the disposable income of consumers in period t. For consumers, non-medical health consumption (*c*_*t*_) and medical health consumption (M_*t*_) are the decision variables of consumers, and consumers can adjust consumption as they wish. Government health investment (*G*_*t*_) is an exogenous variable, mainly based on government budget decisions. The Lagrange function was used to derive the maximum utility function, and the non-medical health consumption (*c*_*t*_) met the following first-order conditions:

(5) $$\begin{array}{l} {\left(1+\rho \right)}^{-t}\alpha {\left(c_{t}^{*}\right)}^{\alpha \left(1-\theta \right)-1}{\left({\text{M}}_{t}\right)}^{\beta (1-\theta )}{\left({\text{G}}_{t}\right)}^{\gamma (1-\theta )}=\text{λ} \end{array}$$

Here, λ is the Lagrange multiplier of the constraint condition, and the natural logarithm is taken on both sides of Equation (5)

(6) $$\begin{array}{l} \ln{c_{t}}^{*}=\frac{\beta \left(1-\theta \right)}{1-\alpha \left(1-\theta \right)}\ln\;M_{t}+\frac{\gamma \left(1-\theta \right)}{1-\alpha \left(1-\theta \right)}lnG_{t}\\ \;+\frac{ln\alpha }{1-\alpha \left(1-\theta \right)}-\frac{t}{1-\alpha \left(1-\theta \right)}\ln\left(1+\rho \right)\\ \;-\frac{1}{1-\alpha \left(1-\theta \right)}ln\lambda \end{array}$$

The Lagrange function is used to derive the maximum utility function, and the medical health consumption (*M*_*t*_) meets the following first-order conditions:

(7) $$\begin{array}{l} {\left(1+\rho \right)}^{-t}\beta {\left(M_{t}^{*}\right)}^{\beta \left(1-\theta \right)-1}{\left({\text{c}}_{t}\right)}^{\alpha \left(1-\theta \right)}{\left({\text{G}}_{t}\right)}^{\gamma \left(1-\theta \right)}=\text{λ} \end{array}$$

Here, λ is the Lagrange multiplier of the constraint condition, and the natural logarithm is taken on both sides of Equation (7) to obtain:

(8) $$\begin{array}{l} \ln{M_{t}}^{*}=\frac{\alpha \left(1-\theta \right)}{1-\beta \left(1-\theta \right)}\ln\;c_{t}+\frac{\gamma \left(1-\theta \right)}{1-\beta \left(1-\theta \right)}lnG_{t}\\ \;+\frac{ln\beta }{1-\beta \left(1-\theta \right)}-\frac{t}{1-\beta \left(1-\theta \right)}\ln\left(1+\rho \right)\\ \;-\frac{1}{1-\beta \left(1-\theta \right)}ln\lambda \end{array}$$

According to Equations (6) and (8), the elasticities of non-medical health consumption and medical health consumption to government health investment are $\frac{\beta \left(1-\theta \right)}{1-\alpha \left(1-\theta \right)}$ and $\frac{\alpha \left(1-\theta \right)}{1-\beta \left(1-\theta \right)}$ respectively. According to α > 0 and β > 0, when consumers have a low degree of relative risk aversion (θ < 1) and a strong preference for medical and health consumption (α < 1/(1−θ), government health investment will increase non-medical health consumption (*c*_*t*_) and medical health consumption (M_*t*_). When the relative risk aversion of consumers is high (θ > 1) and the consumption preferences of residents is weak, government health investment will restrain the growth of the non-health consumption (*c*_*t*_) and health consumption (M_*t*_) of residents. When consumers' relative risk aversion is low (θ < 1) and the preference for medical health consumption is also weak, government health investment will promote non-medical health consumption (*c*_*t*_), but the effect on medical health consumption (M_*t*_) is uncertain. Based on this, there may be a crowding-out effect or crowding-in effect of government health investment on household consumption. Therefore, this paper uses data from inland provinces of China to conduct empirical tests to analyse the impact of government health investment on household consumption.

## Methodology

### Panel Fixed Effects Model

We construct a panel fixed effects model to test the influencing factor of government health investment and household consumption. According to the existing research, the panel fixed effects model formally formulated as follows:

(9) $$\begin{array}{l} C_{it}=\alpha_{0}+\alpha_{1}GHI_{it}+\alpha_{2}X_{it}+\mu_{i}+\varepsilon_{it} \end{array}$$

Here, *GHI*_*it*_ is the government health investment; *C*_*it*_ is the household consumption, including medical health consumption and non-medical health consumption^1^; *X*_*it*_ is the control variables; α_1_ and α_2_ are the coefficients of the variables; μ_*i*_ denotes the fixed effect in different province under varying conditions; ε_*it*_ is a white noise process compliance with $\varepsilon_{it}\sim \left(0,\sigma^{2}\right)$; and *i* and *t* denote the province and time, respectively.

### Sobel-Goodman Mediation Tests

In order to more clearly analyse the path of government health investment on consumer spending, this paper uses the sequential regression test method to construct the mediating effect model as follows:

(10) $$\begin{array}{l} C_{it}=\alpha_{0}+\alpha_{1}GHI_{it}+\alpha_{2}X_{it}+\mu_{i}+\varepsilon_{it} \end{array}$$

(11) $$\begin{array}{l} C_{it}=\beta_{0}+\beta_{1}GHI_{it}+\beta_{2}LGDP_{it}+\beta_{3}X_{it}+\mu_{i}+\varepsilon_{it} \end{array}$$

(12) $$\begin{array}{l} LGDP_{it}=\gamma_{0}+\gamma_{1}GHI_{it}+\gamma_{2}X_{it}+\mu_{i}+\varepsilon_{it} \end{array}$$

where *GHI*_*it*_ is the government health investment; *C*_*it*_ is the household consumption, including medical health consumption and non-medical health consumption; *LGDP*_*it*_ is the logarithm of GDP; *X*_*it*_ is the control variables; α_1_, α_2_, β_1_, β_2_, β_3_, γ_1_ and γ_2_ are the coefficients of the variables; μ_*i*_ denotes the fixed effects in different provinces under varying conditions; ε_*it*_ is a white noise process compliant with $\varepsilon_{it}\sim \left(0,\sigma^{2}\right)$; and *i* and *t* denote the province and time, respectively.

## Data

We used annual data from Chinese inland provinces from 2007 to 2019, with a total of 403 annual terms. Since the government health investment data for most provinces of China were collected starting in 2007, this sample covers a period starting in 2007. The data sources are the National Bureau of Statistics, the China Statistical Yearbook, and the China Financial Statistics Yearbook. Most previous studies believe that household consumption (HC) is the most important indicator to weigh the level of consumption. Medical health care (HCM) and non-medical health consumption (HCN) are separately used to measure the level of consumption. In this paper, government health investment (GHI) is the core explanatory variable. A large number of empirical studies have demonstrated that the level of government health investment has an impact on household consumption. However, government health investment care is also an expensive activity that does not guarantee potential returns, which motivates us to study whether the impact of government health investment on consumer consumption is crowing-in or crowing-out.

Because uncertainty in external factors may affect the relationship of variables (29), this paper introduced seven control variables in this study. The first is the consumer price index (*CPI*), which captures changes in the price levels of the consumer goods and services generally purchased by households. The rate of change in *CPI* reflects the degree of inflation or deflation and individuals will weigh the costs and benefits of spending on consumption capacity. The second control variable is the logarithm of per capita disposable income (*CI*), which is considered the most important determinant of consumer spending. The third is urbanization (*URB*), which is measured by the proportion of the urban population to the total population. The level of urbanization reflects the degree of regional economic development and is an important measure of the degree of regional economic development. Generally, urbanization can promote economic growth by expanding demand (30), and the higher the degree of urbanization is, the higher the consumption level of residents. This means that an increase in the urban proportion of the population can drive a continuous increase in overall consumption power (31, 32). The fourth is the industrial structure (*ISA*), which is measured by the ratio of the output value of the tertiary industry to the secondary industry. The upgrading of the industrial structure refers to the process in which the center of gravity of the industrial structure is gradually transferred from the primary industry to the secondary industry and the tertiary industry, marking the level, stage, and direction of a country's economic development. The fifth is infrastructure (*INFS*), which is measured in terms of total rail mileage per million square kilometers and reflects the development level of railway transportation infrastructure. The sixth is the dependency ratio (*DEP*), which is measured by the sum of the old-age dependency ratio and the young dependency ratio. The larger the dependency ratio is, the greater the number of dependents per worker is, which means the more serious the dependency burden of the labor force is. To a certain extent, upbringing has a certain impact on household consumption. The seventh is the regional average housing price, which is measured by the average price of commercial housing (*HP*). The cost of a house purchase usually accounts for an important proportion of household consumption and has a significant impact on it (33), and through wealth channels and loan restrictions to influence household consumption decisions (34).

**Figure 2:**
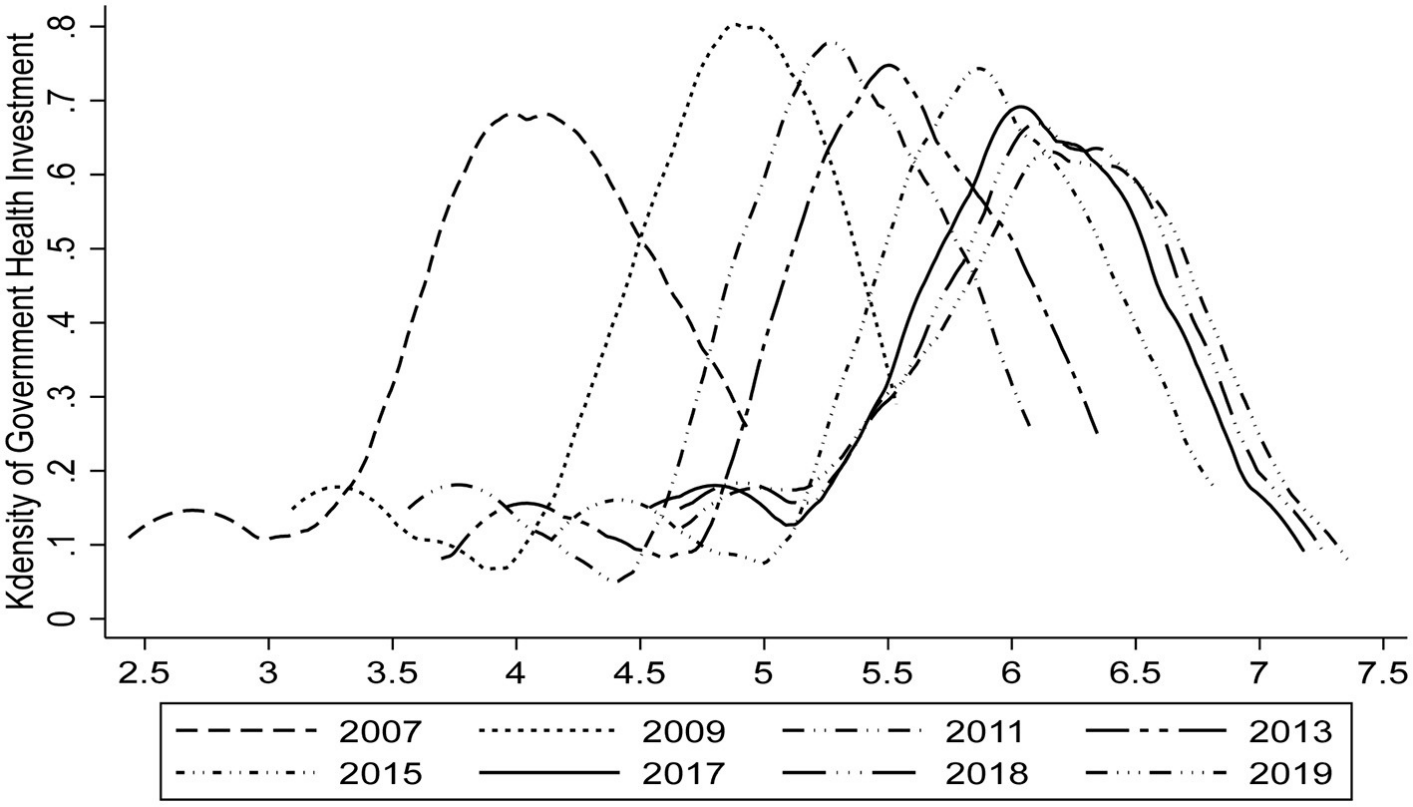
Kdensity plot of government health investment.

Figure 2 shows the kernel density plot of government health investment in different years. As Figure 2 shows, the nuclear density curve continues to move to the right by year, and the peak value first increases and then decreases. It has basically stabilized since 2017 with 6 units on the abscissa and 7 units on the ordinate, which intuitively reflects the government health investment in recent years presents an upward trend.

**Figure 3:**
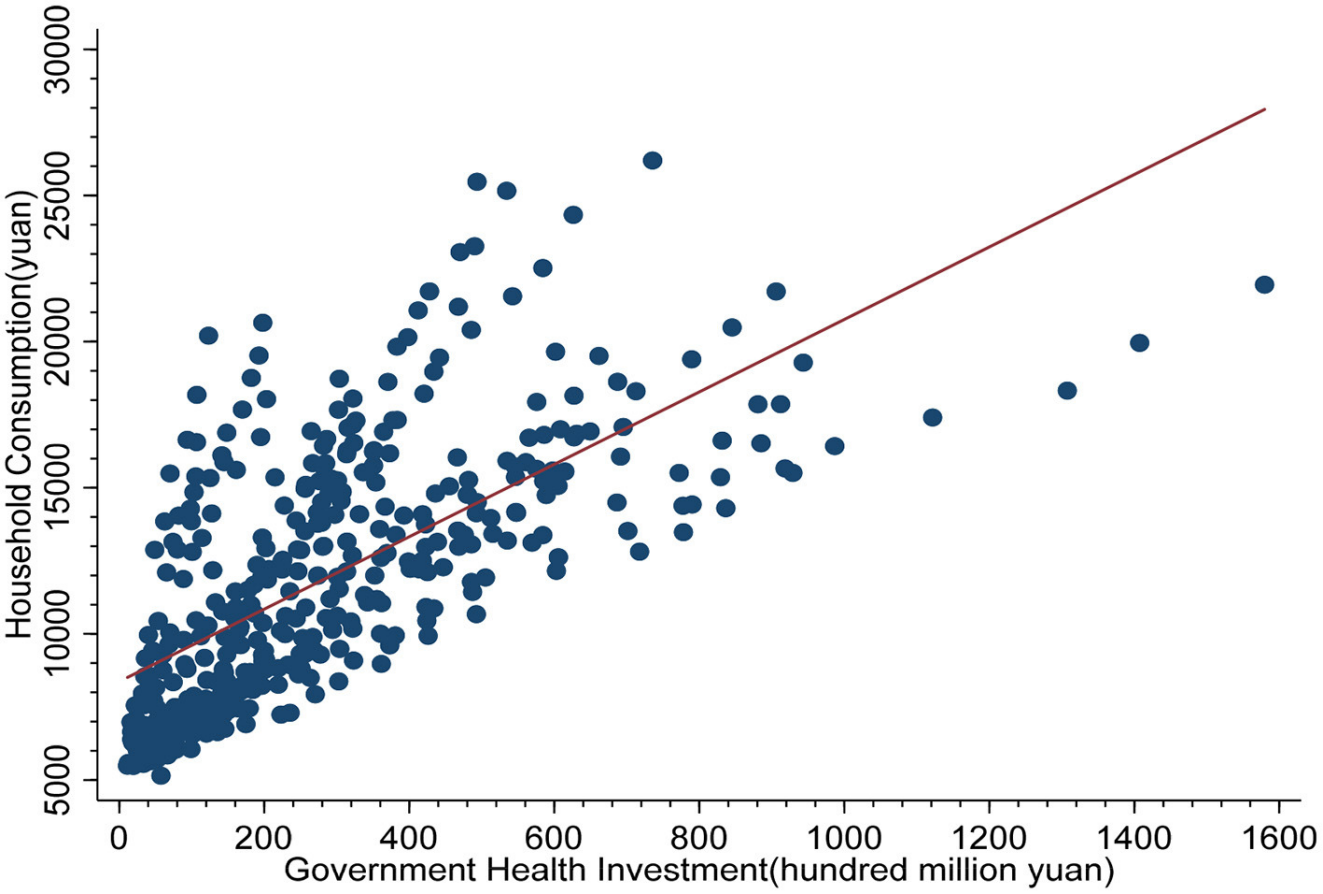
Scatter plot of government health investment and household consumption.

Figure 3 shows the scatter plot of government health investment and household consumption with the abscissa as government health investment and the ordinate as household consumption. As Figure 3 shows, the distribution of government health investment and household consumption is concentrated on the left side of the coordinate axis, and there is a linear fitting relationship, indicating that there is a certain degree of correlation between them.

Table 1 presents the statistical description of the variables. As Table 1 shows, government health investment has a higher mean value of 9.320 than medical health care at 6.607 and non-medical health consumption at 9.289. This may be related to the policy of expanding health investment in China. However, in fact, the difference between government health investment and non-medical health consumption is not obvious, that is, government health investment has been basically equal to residents' non-medical health consumption. Regarding the consumer price index, during the research period, China's price change range was small and basically stable. However, disposable income changed greatly, with a difference of 43.358 units between the minimum value (24.898) and the maximum value (68.256), with the rate of change being as high as 174%. Additionally, the dependency ratio has a high rate of change, and the data structure is skewed to the right. By contrast, the change in housing prices is relatively small, which may be related to local government policies to control housing prices.

**Table 1:** Descriptive statistics of the variables.

	**Obs**	**Mean**	**Std.Dev**.	**Minimum**	**Maximum**	**Skewness**	**Kurtosis**
HC	403	9.320	0.372	8.546	10.173	0.738	0.000
HCM	403	6.607	0.568	5.155	10.259	0.000	0.000
HCN	403	9.289	0.531	8.090	10.647	0.927	0.004
GHI	403	5.310	0.914	2.435	7.365	0.000	0.968
CPI	403	102.846	1.857	97.650	110.090	0.000	0.020
CI	403	41.807	6.957	24.898	68.256	0.016	0.350
URB	403	54.284	14.098	21.500	89.600	0.000	0.071
ISA	403	1.097	0.618	0.500	5.169	0.000	0.000
INFS	403	2.383	1.955	0.043	10.489	0.000	0.000
DEP	403	36.493	6.666	17.825	55.090	0.514	0.017
HP	403	8.604	0.542	7.580	10.489	0.000	0.008

## Empirical Results

Referring to Shiller and Perron (35), the one-equation ADF test is not significant in these samples. In this paper, the Levin, Lin and Chu [(36), LLC] and Im, Pesaran and Shin [(37), IPS] tests are used to assess the unit root problem. As Table 2 shows, there is no unit root of government health investment and household consumption. Additionally, we should confirm the stationary of all variables before using fixed effect analysis to avoid using a pseudoregression. The panel unit root tests for both LLC and IPS show that the variables are all significant at the 10% level. Therefore, we proceed to the regression analysis.

**Table 2:** Panel unit root tests.

**Variables**	**Panel augmented dickey-fuller test**			
	**Levin-Lin-Chu**		**Im-Pesaran-Shin**	
	***t-*statistic**	***p***	***t-*statistic**	***p***
HC	−6.162	0.000	−2.565	0.005
HCM	−43.884	0.000	−13.571	0.000
HCN	−15.121	0.000	−5.235	0.000
GHI	−16.948	0.000	−10.283	0.000
CPI	−2.688	0.004	−16.134	0.000
CI	−4.286	0.000	−36.369	0.000
URB	−19.889	0.000	−8.278	0.000
ISA	−15.468	0.000	−2.480	0.007
INFS	−16.240	0.000	−3.532	0.000
DEP	−19.684	0.000	−21.470	0.000
HP	−20.080	0.000	−10.340	0.000

### Regression Analysis Results

The results recorded, highlighted in Table 3, present an optimal level of household consumption structure. As Table 3 shows, we take government health spending as the core explanatory variable. The regression results of Model 1 and Model 2 show that there is a positive correlation between government health investment and household consumption; and when a series of control variables are added, the regression coefficient between the two decreases from 0.5 to 0.304. However, the regression coefficients of the equations before and after the addition of control variables are significantly positive at least at the 10% significance level. In addition to the core explanatory variables, residents' disposable income, urbanization level, industrial structure, and regional average housing price are also positively correlated with household consumption at least at a 5% significance level. Then, medical health consumption and non-medical health consumption were taken as explained variables for regression. The results of Models 3–6 showed that government health investment was significantly positively correlated with medical health consumption and non-medical health consumption. The regression coefficients also decreased after adding control variables, which were all lower than the regression coefficients of Model 1 (0.304). According to Table 3, the increase in government health investment can significantly improve household consumption.

**Table 3:** Regression analysis results.

**Variables**	**HC**		**HCM**		**HCN**	
	**Model 1**	**Model 2**	**Model 3**	**Model 4**	**Model 5**	**Model 6**
GHI	0.500^***^ (0.015)	0.304^***^ (0.033)	0.368^***^ (0.026)	0.202^**^ (0.098)	0.597^***^ (0.015)	0.285^***^ (0.036)
CPI		0.002 (0.002)		−0.020 (0.014)		0.001 (0.002)
CI		0.006^***^ (0.002)		−0.002 (0.005)		0.006^***^ (0.002)
URB		0.008^**^ (0.004)		0.023^**^ (0.010)		0.025^***^ (0.005)
ISA		0.113^**^ (0.051)		0.170^**^ (0.081)		0.070 (0.070)
INFS		0.036 (0.039)		−0.018 (0.043)		−0.012 (0.021)
DEP		0.004 (0.003)		0.010 (0.006)		0.003 (0.003)
HP		0.181^**^ (0.071)		−0.116 (0.173)		0.271^**^ (0.103)
Constant	6.664^***^ (0.080)	4.841^***^ (0.443)	4.651^***^ (0.136)	6.934^**^ (2.542)	6.120^***^ (0.080)	3.553^***^ (0.667)
N	403	403	403	403	403	403
*R-sq*(overall)	0.9169	0.9621	0.4486	0.4868	0.9018	0.9386

*Cluster-robust standard errors in parentheses.*

^*^Significant at the 10% level.

** *Significant at the 5% level.*

*** *Significant at the 1% level*.

### Heterogeneity Analysis

Table 4 divides the sample into urban residents and rural residents. As Table 4 shows, for rural residents, there is a positive correlation between government health investment and household consumption, medical health consumption, and non-medical health consumption. The regression coefficient of medical health consumption (0.516) is much higher than that of the other two (0.349 and 0.342). For urban residents, there is a significant positive correlation between government health investment and household consumption and non-medical health consumption with regression coefficients of 0.268 and 0.281, respectively; however, the correlation between government health investment and urban medical health consumption is not significant. Overall, the regression coefficient of rural residents (0.349) is higher than that of urban residents (0.268), which means that the impact of government health investment on urban household consumption is less than that on rural household consumption. This may be because in recent years, with the promotion of the government's Medicaid policy, rural residents continue to reduce their medical health consumption; furthermore, government health investment promotes rural non-medical consumption and medical consumption, thus improving rural non-medical health consumption and overall rural household consumption. However, the consumption of urban residents is weaker than that of rural residents due to lack of relevant welfare. In addition, the price index, residents' disposable income, the level of urbanization and the average regional housing price also significantly improve household consumption at least at the 10% significance level.

**Table 4:** Heterogeneity analysis of urban and rural sample.

**Variables**	**Urban sample**			**Rural sample**		
	**HC**	**HCM**	**HCN**	**HC**	**HCM**	**HCN**
	**Model 1**	**Model 2**	**Model 3**	**Model 4**	**Model 5**	**Model 6**
GHI	0.268^***^ (0.035)	0.018 (0.120)	0.281^***^ (0.036)	0.349^***^ (0.051)	0.516^***^ (0.057)	0.342^***^ (0.054)
CPI	0.003^*^ (0.002)	−0.028^*^ (0.016)	0.004^**^ (0.002)	−0.002 (0.002)	0.008^**^ (0.004)	−0.003 (0.002)
CI	0.006^***^ (0.002)	−0.008 (0.006)	0.006^***^ (0.001)	0.007^***^ (0.002)	0.008^***^ (0.003)	0.006^**^ (0.002)
URB	0.009^***^ (0.003)	−0.006 (0.011)	0.005 (0.005)	0.041^***^ (0.007)	0.039^***^ (0.006)	0.040^***^ (0.007)
ISA	0.074 (0.048)	0.186^*^ (0.109)	0.046 (0.057)	0.189^**^ (0.770)	0.120^*^ (0.065)	0.195^**^ (0.082)
INFS	−0.009 (0.188)	0.050 (0.452)	−0.005 (0.005)	0.051 (0.053)	0.067 (0.425)	0.050 (0.055)
DEP	0.002 (0.002)	0.008 (0.007)	0.002 (0.002)	0.007 (0.005)	0.001 (0.004)	0.008 (0.005)
HP	0.297^***^ (0.055)	−0.206 (0.218)	0.328^***^ (0.800)	−0.012 (0.135)	0.146 (0.099)	−0.037 (0.142)
Constant	4.510^***^ (0.340)	11.338^***^ (2.910)	4.267^***^ (0.522)	4.191^***^ (0.788)	−1.285^**^ (0.593)	4.422^***^ (0.845)
N	403	403	403	403	403	403
*R-sq*	0.9717	0.0372	0.8845	0.9407	0.9573	0.9332

*Cluster-robust standard errors in parentheses.*

* *Significant at the 10% level.*

** *Significant at the 5% level.*

*** *Significant at the 1% level*.

There are obvious differences in economic development between the northern and southern regions of China, and the gap has widened. The Huanghe River and Qinling Mountains are the natural geographical boundaries between the northern and southern regions across the areas of Hubei, Shaanxi, and Henan Provinces. Therefore, Table 5 divides the entire sample into two regions: the northern and southern regions. Table 5 shows that in both the south and north, government health investment has a significant promoting effect on household consumption and non-health consumption; and the regression coefficients are 0.332 and 0.306 and 0.288 and 0.259, respectively. However, government health investment has no significant effect on promoting health consumption. In addition to disposable income and the urbanization level, there are significant positive correlations between the industrial structure and housing prices, residents' consumption, and non-medical and health consumption, but there is no such effect in the southern region.

**Table 5:** Heterogeneity analysis of different economic regions sample.

**Variables**	**North region**			**South region**		
	**HC**	**HCM**	**HCN**	**HC**	**HCM**	**HCN**
	**Model 1**	**Model 2**	**Model 3**	**Model 4**	**Model 5**	**Model 6**
GHI	0.332^***^ (0.064)	0.201 (0.179)	0.306^***^ (0.052)	0.288^***^ (0.041)	0.150 (0.099)	0.259^***^ (0.050)
CPI	0.002 (0.003)	−0.033 (0.033)	0.001 (0.002)	0.004^**^ (0.002)	−0.011 (0.009)	0.001 (0.002)
CI	0.006^***^ (0.001)	−0.002 (0.006)	0.006^***^ (0.001)	0.009^**^ (0.003)	−0.001 (0.008)	0.008^*^ (0.004)
URB	−0.002^***^ (0.260)	0.015 (0.023)	0.020^***^ (0.004)	0.018^***^ (0.003)	0.031^***^ (0.008)	0.032^***^ (0.007)
ISA	0.124^***^ (0.026)	0.207 (0.120)	0.107^***^ (0.027)	0.058 (0.070)	0.026 (0.093)	−0.015 (0.109)
INFS	0.054 (0.038)	−0.062 (0.056)	−0.015 (0.023)	0.029 (0.019)	0.090^**^ (0.042)	0.016 (0.026)
DEP	−0.002 (0.004)	0.012 (0.015)	−0.001 (0.003)	0.009^**^ (0.004)	0.011^***^ (0.003)	0.007^**^ (0.003)
HP	0.257^**^ (0.105)	−0.055 (0.283)	0.306^***^ (0.074)	0.100 (0.080)	−0.142 (0.193)	0.226 (0.149)
Constant	4.763^***^ (0.475)	8.321 (5.258)	3.601^***^ (0.277)	4.746^***^ (0.659)	5.894^***^ (1.759)	3.439^***^ (1.141)
N	195	195	195	208	208	208
*R-sq*	0.9617	0.3743	0.9759	0.9750	0.6665	0.9109

*Cluster-robust standard errors in parentheses.*

* *Significant at the 10% level.*

** *Significant at the 5% level.*

*** *Significant at the 1% level*.

Based on the classification of the China Statistics Bureau, all provinces of China are divided into three major economic gradients. There is an obvious development gap in the economic gradients of east, central and west. Table 6 shows that in all economic gradients, government health investment has a significant promoting effect on household consumption and non-health consumption. The regression coefficients are 0.300 and 0.292 in the east gradient, 0.288 and 0.312 in the central gradient, and 0.264 and 0.245 in the west gradient, respectively. However, government health investment has no significant effect on promoting health consumption. In east and central gradients, except for the significant positive correlation between industrial structure and household consumption and non-medical health consumption, all other control variables have no significant relationship with them. But there is no such effect in the west gradient, where per capita disposable income, urbanization, and infrastructure contribute a lot.

**Table 6:** Heterogeneity analysis of different economic gradients sample.

**Variables**	**East gradient**			**Central gradient**			**West gradient**		
	**HC**	**HCM**	**HCN**	**HC**	**HCM**	**HCN**	**HC**	**HCM**	**HCN**
	**Model 1**	**Model 2**	**Model 3**	**Model 4**	**Model 5**	**Model 6**	**Model 7**	**Model 8**	**Model 9**
GHI	0.300^***^ (0.038)	0.231* (0.122)	0.292^***^ (0.048)	0.288^***^ (0.064)	0.273 (0.209)	0.312^***^ (0.057)	0.264^***^ (0.046)	0.125 (0.129)	0.245^***^ (0.061)
CPI	0.001 (0.002)	−0.017 (0.013)	0.001 (0.002)	0.008^*^ (0.004)	−0.056 (0.060)	0.008^**^ (0.003)	0.000 (0.001)	0.004 (0.009)	−0.002 (0.002)
CI	0.003 (0.004)	0.001 (0.008)	0.007^**^ (0.003)	0.003 (0.003)	−0.029 (0.020)	0.003 (0.003)	0.009^***^ (0.002)	0.007 (0.005)	0.008^***^ (0.001)
URB	0.009 (0.006)	0.020^*^ (0.010)	0.024^***^ (0.004)	−0.003 (0.010)	−0.005 (0.025)	0.016 (0.009)	0.027^***^ (0.007)	0.023 (0.024)	0.041^**^ (0.015)
ISA	0.200^***^ (0.059)	0.130 (0.125)	0.191^***^ (0.038)	0.167^***^ (0.018)	0.598^**^ (0.244)	0.126^***^ (0.025)	−0.013 (0.071)	0.060 (0.106)	−0.100 (0.089)
INFS	0.098^*^ (0.049)	0.069 (0.056)	0.028 (0.019)	0.075 (0.068)	−0.085 (0.157)	0.071 (0.069)	−0.100^***^ (0.030)	0.130 (0.133)	−0.103^**^ (0.034)
DEP	0.003 (0.005)	0.009^*^ (0.004)	0.001 (0.003)	0.013^*^ (0.005)	0.041 (0.029)	0.010^*^ (0.005)	0.006 (0.004)	0.001 (0.006)	0.007 (0.004)
HP	−0.015 (0.066)	−0.362 (0.280)	0.068 (0.048)	0.256^*^ (0.120)	−0.212 (0.313)	0.177 (0.108)	0.138^*^ (0.070)	0.168 (0.187)	0.280 (0.177)
Constant	6.386^***^ (0.543)	8.290^***^ (2.388)	5.035^***^ (0.324)	3.988^***^ (0.421)	12.183 (8.400)	3.549^***^ (0.341)	5.088^***^ (0.457)	2.618 (1.641)	3.465^**^ (1.186)
N	143	143	143	104	104	104	156	156	156
*R-sq*	0.9648	0.5658	0.9810	0.9740	0.3737	0.9834	0.9734	0.6969	0.9029

*Cluster-robust standard errors in parentheses.*

* *Significant at the 10% level.*

** *Significant at the 5% level.*

*** *Significant at the 1% level*.

### Robustness Check

In this paper, we adopted a strict regression model. Then, a series of robustness tests were conducted to further ensure the reliability and stability of the study.

The lag term of the explained variable is introduced into the regression model as the explanatory variable in the dynamic panel model, which has dynamic explanatory ability but inevitably results in endogeneity problems. In order to solve the endogeneity problem of the model, this paper uses the difference GMM method proposed by Arellano-Bond for the robustness test (38). Table 7 reports the relationship between government health investment and household consumption in the new period. As Table 7 shows, the regression coefficients of government health investment in model 1 and model 3 are 0.118 and 0.262, respectively, which are significant at the 1% level; furthermore, the coefficient of government health investment in model 2 is 0.314, which is significantly positive at the 5% level. This indicates that when the lagged order of the original equation variable is used as the instrumental variable, the above results are still valid, that is, government health investment has a significant promoting effect on household consumption.

**Table 7:** Robustness check.

**Variables**	**Gaussian mixture model**			**Change time period (2009–2019)**		
	**HC**	**HCM**	**HCN**	**HC**	**HCM**	**HCN**
	**Model 1**	**Model 2**	**Model 3**	**Model 4**	**Model 5**	**Model 6**
GHI	0.118^***^ (0.041)	0.314^**^ (0.143)	0.262^***^ (0.068)	0.412^***^ (0.039)	0.293^**^ (0.121)	0.373^***^ (0.053)
CPI	0.005^***^ (0.002)	−0.017 (0.013)	0.003^*^ (0.119)	−0.014^***^ (0.002)	−0.025 (0.016)	−0.015^***^ (0.002)
CI	−0.001 (0.005)	−0.006 (0.012)	0.003 (0.003)	0.005^***^ (0.001)	−0.004 (0.006)	0.004^***^ (0.494)
URB	−0.001 (0.004)	0.027 (0.021)	0.018^***^ (0.006)	0.001 (0.004)	0.019 (0.012)	0.020^***^ (0.005)
ISA	−0.007 (0.036)	0.124 (0.186)	0.071 (0.055)	0.055^**^ (0.026)	0.152 (0.108)	0.024 (0.032)
INFS	0.007 (0.033)	−0.050 (0.148)	−0.017 (0.034)	0.038 (0.026)	−0.015 (0.059)	−0.007 (0.014)
DEP	0.004 (0.004)	0.026^*^ (0.015)	0.001 (0.550)	−0.007 (−0.320)	0.013 (0.009)	−0.001 (0.002)
HP	0.017 (0.052)	−0.223 (0.184)	0.122 (0.119)	0.256^***^ (0.047)	−0.174 (0.189)	0.342^***^ (0.088)
L.HC	0.765^***^ (0.080)					
L.HCM		−0.127 (0.139)				
L.HCN			0.260 (0.199)			
Constant				6.013^***^ (0.271)	7.655^***^ (2.608)	4.708^***^ (0.494)
AR(1)	0.000	0.108	0.326	—	—	—
AR(2)	0.459	0.462	0.407	—	—	—
Hansen	1.000	1.000	1.000			
N	341	341	341	341	341	341

*Cluster-robust standard errors in parentheses.*

* *Significant at the 10% level.*

** *Significant at the 5% level.*

*** *Significant at the 1% level*.

The research above uses the statistical data of China's inland provinces from 2007 to 2019 as samples for analysis. However, due to the government's opinions on deepening the reform of the medical and health system in 2009 and considering the lag effects of policy reform and government health investment on household consumption, the data of the previous 2 years are excluded, and the research period is from 2009 to 2019. Table 7 reports the relationship between government health investment and household consumption in the new period. Since the coefficients of government health investment in models 4–6 are 0.421, 0.293 and 0.373, respectively, and they are significantly positive at least at the 5% level, the above estimation results are still robust in different research periods.

## Mechanism Analysis

The previous theory analyses the transmission mechanism between government health investment and household consumption. In order to test the hypothesis of the mechanism, this paper selects the mediating effect model for empirical testing, and the regression results are shown in Table 8. Model 1 shows that there is a positive correlation between government health investment and per capita GDP, and it is significantly positive at the 1% level, which indicates that government health input can promote economic growth, which is embodied in the increased per capita GDP. Similarly, after adding the mediating variable, the government health investment variable is no longer significant, and the mediating variable is significantly positive at the 1% level, which indicates that there is a complete mediating effect in per capita GDP. That is, government health investment boosts regional economic growth, which in turn boosts household consumption. In order to explore the mechanism of government health investment on residents' non-medical health consumption and medical health consumption more clearly, we also test the mediating effect. The results show that government health investment can promote non-medical health consumption by accelerating economic growth. As Models 3, 5, and 7 show, the government health investment variable is no longer significant after adding the mediating variable. Furthermore, per capita GDP, as the mediating variable, is significantly positive at the 1% significance level with coefficients of 0.728, 0.377, and 0.996, respectively, indicating that per capita GDP has a complete mediating effect. Government health investment can promote household consumption by increasing the speed of economic development (39). In order to explore the mechanism of the effect government health investment more clearly on residents' non-medical health consumption and medical health consumption, we tested its mediating effect, and the results show that government health investment can stimulate non-health consumption by increasing the economic growth rate, and this stimulating effect does not reduce medical consumption.

**Table 8:** Mechanism analysis of government health investment and household consumption.

**Variables**	**LPGDP**	**HC**		**HCM**		**HCN**	
	**Model 1**	**Model 2**	**Model 3**	**Model 4**	**Model 5**	**Model 6**	**Model 7**
LPGDP			0.728^***^ (0.107)		0.377^*^ (2.583)		0.996^***^ (0.185)
GHI	0.329^***^ (0.033)	0.304^***^ (0.033)	0.064 (0.047)	0.202^**^ (0.098)	0.078 (0.105)	0.285^***^ (0.034)	−0.043 (0.075)
CPI	0.007^***^ (0.001)	0.002 (0.002)	−0.003^*^ (0.002)	−0.020 (0.014)	−0.023 (0.015)	0.001 (0.002)	−0.007^***^ (0.002)
CI	−0.015^***^ (0.002)	0.006^***^ (0.002)	0.017^***^ (0.003)	−0.002 (0.005)	0.003 (0.006)	0.006^***^ (0.002)	0.021^***^ (0.004)
URB	0.019^***^ (0.004)	0.008^**^ (0.004)	−0.006 (0.004)	0.023^**^ (0.010)	0.016 (0.011)	0.025^***^ (0.005)	0.006 (0.005)
ISA	0.051 (0.048)	0.113^**^ (0.051)	0.075^**^ (0.032)	0.170^**^ (0.081)	0.161^*^ (0.076)	0.070 (0.070)	0.019 (0.047)
INFS	−0.028 (0.017)	0.036 (0.039)	0.056 (0.043)	−0.018 (0.043)	−0.008 (0.040)	−0.012 (0.021)	0.015 (0.023)
DEP	0.001 (0.002)	0.004 (0.003)	0.004 (0.002)	0.010 (0.006)	0.010 (0.006)	0.003 (0.003)	0.003^*^ (0.001)
HP	0.295^***^ (0.042)	0.181^**^ (0.071)	−0.034 (0.050)	−0.116 (0.173)	−0.227 (0.194)	0.271 (0.103)	−0.023 (0.055)
Constant	5.078^***^ (0.290)	4.841^***^ (0.443)	1.143 (0.714)	6.934^**^ (2.542)	5.023^*^ (2.583)	3.553^***^ (0.667)	−1.505 (1.315)
N	403	403	403	403	403	403	403
*R-sq*	0.9790	0.9621	0.9759	0.4868	0.4901	0.9386	0.9565

*Cluster-robust standard errors in parentheses.*

* *Significant at the 10% level.*

** *Significant at the 5% level.*

*** *Significant at the 1% level*.

## Conclusions

This paper performs a panel unit root test to examine government health investment and household consumption. Table 2 shows that the stationary hypothesis of household consumption is undeniable. Household consumption follows a random distribution, and government health investment is an important variable that affects household consumption. Thus, based on the panel data of China's inland provinces from 2007 to 2019, we use a regression model to explore the crowding-out or crowding-in relationship between Chinese government health investment and household consumption. The empirical results above show that government health investment has a crowding-in effect, which can raise household consumption. In our study, household consumption is divided into all household consumption, medical health consumption, and non-medical health consumption. Based on northern and southern provinces, there is a significant positive correlation between government health investment and urban and rural household consumption, and the promotion effect on the northern provinces is better than that on the southern provinces; however, there is no significant positive correlation between government health investment and medical health consumption in some regions. In addition, this paper uses the logarithm of per capita GDP as an intermediary variable to test the mechanism between government health investment and household consumption. The results show that per capita GDP has a complete mediating effect. That is, government health investment boosts regional economic growth, which in turn increases household consumption. These conclusions provide valuable insights for decision makers in the health care sector to intervene when facing drastic economic changes. The government should adopt more active medical policies and health investment policies to reduce medical health consumption, increase non-medical health consumption and promote household consumption.

## Data Availability Statement

The original contributions presented in the study are included in the article/supplementary material, further inquiries can be directed to the corresponding author/s.

## Author Contributions

HC: conceptualization and methodology. Y-PZ: software and writing-original draft preparation. Z-WD: data curation and writing-reviewing. QG: visualization and editing. RJ: investigation. All authors contributed to the article and approved the submitted version.

## Conflict of Interest

The authors declare that the research was conducted in the absence of any commercial or financial relationships that could be construed as a potential conflict of interest.

## Footnotes

^1^According to the classification of China Statistical Yearbook, the consumption expenditure of residents can be divided into two categories: medical consumption and non-medical consumption. Non-medical expenditure includes food research, clothing, living, living goods service, traffic information, education, culture and entertainment, and other goods services, and medical treatment expenditure includes medical and health care expenditure.
